# Air toxics and early childhood acute lymphocytic leukemia in Texas, a population based case control study

**DOI:** 10.1186/s12940-016-0154-8

**Published:** 2016-06-14

**Authors:** Elaine Symanski, P. Grace Tee Lewis, Ting-Yu Chen, Wenyaw Chan, Dejian Lai, Xiaomei Ma

**Affiliations:** Department of Epidemiology, Human Genetics and Environmental Sciences, University of Texas Health Science Center at Houston School of Public Health, Houston, Texas USA; Department of Biostatistics, University of Texas Health Science Center at Houston School of Public Health, Houston, Texas USA; Yale University School of Public Health, New Haven, Connecticut USA

**Keywords:** Air toxics, Benzene, 1,3-Butadiene, Polycyclic organic matter, POM, Childhood leukemia, Epidemiology, Acute lymphocytic leukemia, Childhood cancer

## Abstract

**Background:**

Traffic exhaust, refineries and industrial facilities are major sources of air toxics identified by the U.S. Environmental Protection Agency (U.S. EPA) for their potential risk to human health. In utero and early life exposures to air toxics such as benzene and 1,3-butadiene, which are known leukemogens in adults, may play an etiologic role in childhood leukemia that comprises the majority of pediatric cancers. We conducted a population based case–control study to examine individual effects of benzene, 1,3-butadiene and polycyclic organic matter (POM) in ambient residential air on acute lymphocytic leukemia (ALL) diagnosed in children under age 5 years in Texas from 1995–2011.

**Methods:**

Texas Cancer Registry cases were linked to birth records and then were frequency matched by birth month and year to 10 population-based controls. Maternal and infant characteristics from birth certificates were abstracted to obtain information about potential confounders. Modelled estimates of benzene, 1,3-butadiene and POM exposures at the census tract level were assigned by linking geocoded maternal addresses from birth certificates to U.S. EPA National-Scale Air Toxics Assessment data for single and co-pollutant statistical analyses. Mixed-effects logistic regression models were applied to evaluate associations between air toxics and childhood leukemia.

**Results:**

In adjusted single pollutant models, odds of childhood leukemia among mothers with the highest ambient air exposures compared to those in the lowest quartile were 1.11 (95 % CI: 0.94–1.32) for POM, 1.17 (95 % CI: 0.98–1.39) for benzene and 1.29 (95 % CI: 1.08–1.52) for 1,3-butadiene. In co-pollutant models, odds ratios for childhood leukemia remained elevated for 1,3-butadiene but were close to the null value for benzene and POM.

**Conclusions:**

We observed positive associations between 1,3-butadiene and childhood leukemia in single and co-pollutant models whereas effect estimates from single pollutant models were diminished for benzene and POM in co-pollutant models. Early life exposure to 1,3-butadiene rather than benzene or POM appears to increase early childhood risk of acute lymphocytic leukemia.

**Electronic supplementary material:**

The online version of this article (doi:10.1186/s12940-016-0154-8) contains supplementary material, which is available to authorized users.

## Background

Leukemia is the most common pediatric cancer in the United States (U.S.) [1]. About 80 % of childhood leukemias are acute lymphocytic leukemia (ALL) with approximately 2,200 new cases of ALL diagnosed annually in children under 15 years in the United States [2]. Most childhood ALL is diagnosed between ages 2 and 5 years with higher rates for males than females and for Hispanics and whites as compared to blacks [3]. In Texas, leukemia also leads childhood cancers and accounted for 32.2 % of cases in children 0–14 years from 2007–2011 [4]. Among Texas children 0–4 years, there are on average 119 new ALL diagnosed annually [5].

Aside from ionizing radiation and specific genetic disorders [6–9], the etiology of childhood leukemia, which likely varies by subtype [10], remains largely unknown. There has been growing interest in identifying environmental risk factors that may play a role in pathogenesis and one focus has been on air toxics. Air toxics, also known as hazardous air pollutants, were first identified in 1990 by the U.S. Environmental Protection Agency (EPA) because of concerns about their impact on human health [11]. Emissions from industrial facilities, refineries and power plants, as well as automotive exhaust, are major sources of air toxics [12–15]. Of particular interest are benzene and 1,3-butadiene that are known leukaemogens in adults [11, 16, 17]. Additionally, we focused on polycyclic organic matter (POM) representing a broad range of organic compounds comprised of two to seven fused aromatic rings of which polycyclic aromatic hydrocarbons (PAHs) are the most common subclass [18, 19].

Relatively few investigations of associations between benzene, 1,3-butadiene or PAHs and childhood cancer risk have been conducted and findings have been equivocal. For benzene, six population-based case control studies and one ecologic study have evaluated the association with childhood leukemia [20–25]. Two population based case control studies, one in Italy and the other in California, reported positive associations with estimated relative risks that ranged from 1.50 to 3.91 among children with high benzene exposure [20, 21]. An ecologic study in Texas likewise observed higher odds of childhood leukemia in census tracts with upper quartile ambient benzene levels (OR = 1.37, 95 % CI: 1.05, 1.78) [25]. Four case control studies conducted in Europe, on the other hand, observed null associations [22–24, 26]. Among these investigations, the study populations included children diagnosed at different ages, namely, less than 19 years of age [25], less than 15 years of age [20, 22–24, 26] and less than 5 years of age [21, 24]. Despite its established carcinogenicity in adults [16], 1,3-butadiene studies in children are rare. Two US studies, one a case–control study and the other an ecologic study, have assessed the role of 1,3-butadiene relative to childhood leukemia risk. For children aged 19 years or younger at diagnosis in Southeast Texas who lived in census tracts with the highest 1,3-butadiene levels, elevated rate ratios were detected for all leukemias and ALL [25]. In a more recent investigation of Californian children diagnosed under the age of six years, Heck et al. found elevated odds for both ALL and acute myeloid leukemia (AML) [21]. Among the three population based case–control investigations of PAHs and leukemia risk [21, 27, 28] that also varied in diagnosis ages of children who comprised the study populations, two found positive associations [21, 27] and one reported no association [28]. All were conducted in the US and used modeled or measured estimates of PAH exposures [21, 27, 28].

Owing to the scarcity of studies that have examined a possible etiologic role of air toxics in early childhood leukemia and building on our earlier ecologic investigation of children of all ages [25], we designed a case–control study to examine the hypothesis that risk for ALL among children diagnosed under the age of five increases with exposure to benzene, 1,3-butadiene and PAHs *in utero* and in infancy. This study uses data from one of the largest population-based cancer registries in the U.S., links registry data to birth certificates to obtain information on maternal and infant characteristics and assesses both single and co-pollutant models of three air toxics that are known carcinogens in adults.

## Methods

The study protocol was approved by the Institutional Review Boards at the University of Texas Health Science Center at Houston and the Texas Department of State Health Services (TX DSHS).

### Study population

We obtained incident cases of ALL and AML diagnosed from January 1, 1995 to October 31, 2011 from the Texas Cancer Registry. Our study population was limited to children diagnosed under age 5 years regardless of race or ethnicity. We used the Surveillance, Epidemiology and End Results Program (SEER) recode of the International Classification of Diseases for Oncology, 3rd Edition (ICD-O-3), World Health Organization (WHO) 2008 codes (2/9/2001 update) by histology type to define ALL (codes: 9826, 9835–9837) and AML (codes: 9840, 9861, 9865–9867, 9869, 9871–9874, 9891, 9895–9897, 9898, 9910–9911, 9920) cases. Only cases with birth records that could be linked to the Texas Cancer Registry were eligible for inclusion. Controls were sampled from TX DSHS vital statistics birth records from 1991 to 2009. A 10:1 matching ratio was used to select controls based on birth year and month to allow for the same potential for exposures to air toxics. For all birth records, geocoded addresses of maternal residence at delivery were obtained and records were linked to the TX DSHS Birth Defects Registry to identify infants with congenital anomalies. In total, 1,741 leukemia cases and 17,410 population based controls were identified. We excluded 2,025 birth records with missing geocoding data, 428 non-singleton births and 119 infants born with one or more birth defects. After applying exclusion criteria, 16,579 observations remained. Based on statistical power implications and number of AML (*n* = 170) in our study population, we restricted analysis to ALL cases (*n* = 1,248) and matched controls (*n* = 12,172).

### Exposure assessment

**Fig. 1:**
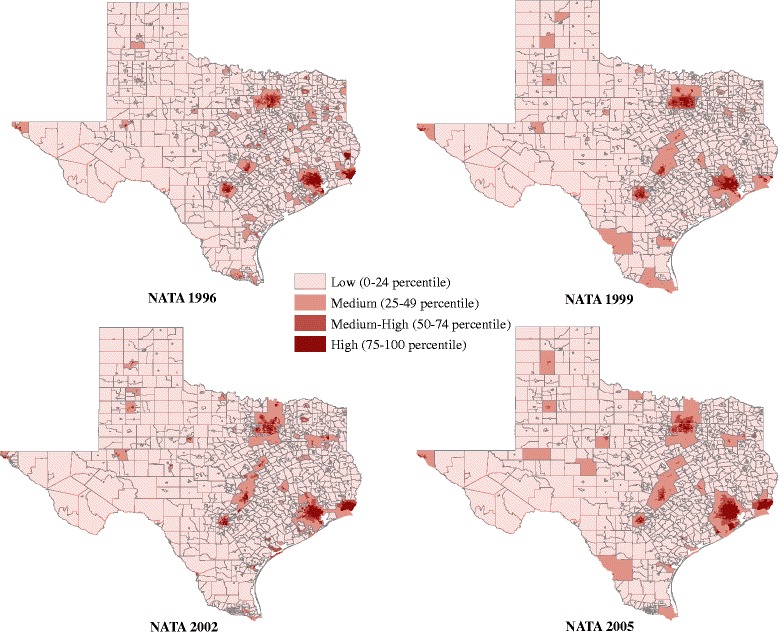
1,3-Butadiene Ambient Air Levels in Texas by NATA year. Exposure groups: Low (0-24^th^ percentile); Medium (25-49^th^ percentile); Medium-High (50–74^th^ percentile); High (75–100^th^ percentile) based on distribution among controls. Boundary lines for census tracts were removed for the Medium, Medium-High, and High groups and graduated color used instead to improve readability of maps and spatial distribution of exposure quartiles

Our exposure assessment for benzene, 1–3, butadiene and PAHs was based on maternal address at delivery and relied upon data from the U.S. EPA National-Scale Air Toxics Assessment (NATA) [29]. NATA provides modeled estimates of air toxics in specific years (1996, 1999, 2002 and 2005) for all census tracts in the continental US, Puerto Rico and the Virgin Islands using the Assessment System for Population Exposure Nationwide (ASPEN). ASPEN uses annual National Toxic Inventory emission data of hazardous air pollutants from major (point) source, area, monitoring, and emissions as inputs in modelling exposures levels. Emissions from background and mobile sources are also estimated and combined with other inputs to produce census tract level HAP modelled estimates [30]. The ASPEN model accounts for the rate, location and height of release of pollutants, wind speed and direction, reactive decay, deposition and secondary formation or decay [30, 31]. Data on benzene, 1,3-butadiene and POM (or PAHPOM as per NATA 2002 and 2005) are available in all NATA releases [18]. POM defines a broad class of compounds that includes the PAHs. We used total POM in our analysis rather than a POM subset of PAHs due to inconsistent availability of PAH groups across NATA data releases [32]. Total POM is available in all NATA years whereas modelled estimates of PAHs are only available in the 1996 and 1999 releases [32]. A map illustrating NATA estimated ambient air levels of 1,3-butadiene for all Texas census tracts corresponding to the release years used in our study is given in Fig. 1. Maps for benzene and POM are available as supplemental figures [see Additional file 1: Figures S1 and Additional file 2: Figure S2, respectively]. Geocoded addresses from birth certificates for infants born in 1991–1997, 1998–2000, 2001–2003 and 2004–2011 were linked to the 1996, 1999, 2002 and 2005 NATA data, respectively.

### Covariates

We abstracted maternal and infant characteristics from birth certificates to evaluate as potential confounders, including maternal age at the time of child’s birth (years), marital status (married, not married) and trimester prenatal care started (first, second, or third). Maternal race and ethnicity were categorized as: white, non-Hispanic; black, non-Hispanic; Hispanic; other. Because the TX DSHS updated birth certificates in 2005, we standardized coding for maternal smoking during pregnancy (yes/no) and educational level (elementary: 0-6th grade, before high school: 7-11th grade, high school: 12th grade, Associate’s degree and Bachelor’s degree and higher). Birth weight (grams), sex and length of gestation (full term, 37 completed weeks or more of gestation; mildly preterm, 32–36 weeks gestation; and severely preterm, < 32 weeks gestation) were also evaluated as confounders. Finally, based on the U.S. 2000 Census, percent of people living below the poverty level was assessed as a potential neighborhood-level covariate in the analyses [33].

### Statistical methods

#### Single and co-pollutant models

Mixed-effects logistic regression models were applied to evaluate the associations between air toxics and childhood leukemia using PROC GLIMMIX in SAS statistical software (version 9.3, SAS Inc., Cary, NC). Air pollutants were modeled categorically using the distribution among the controls for each NATA year to define quartile cut points (low, medium, medium-high and high). The lowest quartile was the referent category. For each air pollutant, we ran models adjusting for the matching factors [34, 35] (infant’s birth year and month) and a random effect for census tract (hereafter referred to as Model 1). To evaluate confounding, each potential covariate was added to Model 1 and the percentage of change in the magnitude of the odds ratio for each air pollutant was calculated between models. While none of the covariates selected for analysis changed the estimated odds ratios by 10 % or more [36], we further applied a backward model selection method with a significance level set at 0.05 to account for the potential for joint confounding and obtained the final regression equation. Six observations were dropped from models due to missing maternal age and infant birth weight. For all three air toxics, we obtained a model that included mother’s age and race/ethnicity and infant’s birth weight and gender (hereafter referred to as Model 2). We also included two air toxics together in co-pollutant models (benzene and 1,3-butadiene; benzene and POM; 1,3 butadiene and POM) and assessed potential confounding effects of these three pollutants by comparing to the results from the single pollutant models.

#### Correlations and collinearity diagnostics

Correlations between categorical rankings of pollutants were evaluated using Spearman’s rank correlation coefficient: ρ = 0.81 for benzene and 1,3-butadiene; ρ = 0.74 for benzene and POM; and ρ = 0.75 for 1,3-butadiene and POM). Given the extent of correlation using quartile exposure groupings, we performed additional diagnostics for collinearity [37]. We used PROC GENMOD in SAS to generate information matrices and weights for the information matrices. Using these outputs for model iterations, we applied SAS PROC REG with VIF and COLLIN options to assess collinearity and found no evidence of collinearity in categorical forms of our exposure variables. VIF values in our collinearity diagnostics ranged from 4.0 to 5.5 and well below VIF ≥ 10.0 rule of thumb [38]. Further, conditional index numbers were all less than 4.0 and well below the cut point of 30 indicative of potential collinearity [38].

### Sensitivity analyses

We conducted a sensitivity analysis limiting our study population to births within a year before or after the NATA data release year: 1996 (1995–1997 births), 1999 (1998–2000 births), 2002 (2001–2003 births), and 2005 (2004–2006 births). Further, recognizing possible spatial correlations of residuals, we also conducted a sensitivity analysis exploring a different error structure for single pollutant models using SAS Proc GLIMMIX to account for exponential spatial correlation. We deleted 167 records with duplicate longitude and latitude coordinates and assumed the spatial correlations were constrained within year and month to address computational constraints. We found little differences in the point estimates and standard errors in comparing results to the mixed effects models (with census tract specified as a random effect) and, hence, report on results using the more parsimonious model.

## Results

Maternal and infant characteristics of the study population at the time of delivery appear in Table 1. Most mothers were married, did not smoke cigarettes during pregnancy and lived in neighborhoods with poverty rates under 20 %. Based on results of crude logistic regression models for each maternal or infant characteristic, there were differences at delivery in maternal age, marital status, and race/ethnicity, infant birth weight and gender for cases as compared to controls.

**Table 1:** Maternal and Infant Characteristics of Childhood Acute Lymphocytic Leukemia Cases and Controls at Birth in Texas, 1995-2011

Characteristic	Cases	Controls	Crude OR^a^ (95 % CI)
	*N* = 1,248	*N* = 12,172	
Maternal Age (years) (Mean (SD))	26.8 (6.2)	26.1 (6.0)	1.02 (1.01, 1.03)
Birth weight (grams) (Mean (SD))	3411.6 (525.1)	3309.7 (549.7)	1.04^b^ (1.03, 1.05)
Infant Gender (%)			
Male	55.5	52.0	1.15 (1.02, 1.30)
Female	44.5	48.0	1.00
Marital Status (%)			
Married	70.4	66.9	1.00
Not Married	29.6	33.0	0.85 (0.75, 0.97)
Cigarette Smoking during Pregnancy (%)			
Yes	5.4	6.0	0.89 (0.68, 1.15)
No	93.5	93.0	1.00
Percentage Living Below Poverty Level^c^ (%)			
< 20 %	66.2	65.3	1.00
20 %	33.8	34.7	0.96 (0.85, 1.08)
Maternal Education^d^ (%)			
Elementary	29.6	29.4	1.17 (0.89, 1.53)
Before high school	19.3	20.2	1.03 (0.86, 1.24)
High school	21.1	22.7	1.00
Associate degree	14.3	12.7	1.21 (0.99, 1.48)
Bachelor and graduate degree	15.0	13.8	1.17 (0.96, 1.43)
Maternal Race/Ethnicity (%)			
White, Non-Hispanic	40.6	38.4	1.00
Black, Non-Hispanic	5.5	12.4	0.41 (0.32, 0.54)
Hispanic	50.6	45.6	1.05 (0.93, 1.20)
Other	3.3	3.6	0.86 (0.61, 1.20)

^a^Estimates from mixed effects logistic regression model adjusted for the matching variables and census tract (random effect)

^b^ORs reported per 100 gram increase in birth weight

^c^Based on maternal residence. Data source: USA Census 2000; www.census.gov

^d^Highest level completed

**Fig. 2:**
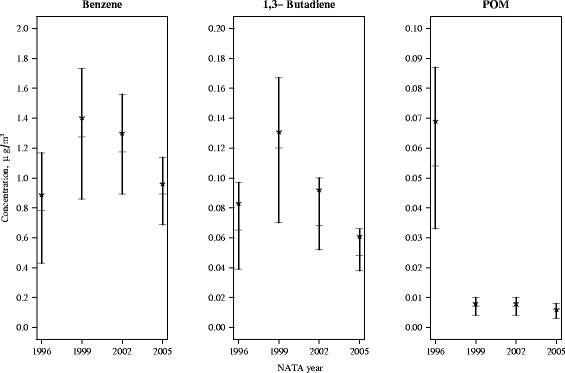
Distribution of Benzene, 1,3-Butadiene, and Polycyclic Organic Matter (POM) for selected percentiles: 25 %, 50 %, 75 % by year for controls (*N* = 12,178), based on maternal address at birth, Texas. Mean denoted by *. Data source: National-Scale Air Toxics (NATA), U.S. Environmental Protection Agency. www.epa.gov/national-air-toxicsassessment

Selected percentiles of the distributions of residential benzene, 1,3-butadiene and POM levels (μg/m^3^) in control subjects, by NATA year, are shown in Fig. 2. Benzene had the highest concentration levels across all years as compared to 1,3-butadiene and POM. The mean (± standard deviation (SD)) concentration of benzene in 1996 was 0.89 (±0.68) μg/m^3^, 1.40 (±0.78) μg/m^3^ in 1999, 1.30 (±0.66) μg/m^3^ in 2002 and 0.96 (±0.46) μg/m^3^ in 2005. Mean levels of 1,3-butadiene were 0.08 (±0.18) μg/m^3^, 0.13 (±0.11) μg/m^3^, 0.09 (±0.12) μg/m^3^ and 0.06 (±0.05) μg/m^3^, in 1996, 1999, 2002 and 2005, respectively, while mean POM concentrations were 0.069 (±0.069) μg/m^3^ in 1995, 0.008 (±0.007) μg/m^3^ in 1999, 0.008 (±0.006) μg/m^3^ in 2002 and 0.006 (±0.004) μg/m^3^ in 2005.

Odds ratios (OR) and 95 % confidence intervals (CI) for benzene, 1–3, butadiene and POM in single pollutant models are given in Table 2. In the fully adjusted model (Model 2) for benzene, we found elevated odds for ALL in the medium (OR = 1.19; 95 % CI: 1.00–1.41), medium-high (OR = 1.16; 95 % CI: 0.98–1.38) and high exposure groups (OR = 1.17; 95 % CI: 0.98–1.39) as compared to the referent group. For 1,3-butadiene, we observed odds ratios of 1.23 (95 % CI: 1.03–1.46), 1.23 (95 % CI: 1.04–1.47) and 1.29 (95 % CI: 1.08–1.52) for the medium, medium-high and high exposure groups, respectively. Albeit not statistically significant, we observed positive associations between ALL and POM for those in the medium-high (OR = 1.18; 95 % CI: 1.00–1.39) and high exposure (OR = 1.11; 95 % CI: 0.94–1.32) categories.

**Table 2:** Association between Childhood Acute Lymphocytic Leukemia in Texas and Air Toxics: Benzene, 1,3-Butadiene, and Polycyclic Organic Matter using Single Pollutant Models

Air Toxic	Model 1^a^ OR (95 % CI)	Model 2^b^ OR (95 % CI)
Benzene		
Low	1.00	1.00
Medium	1.18 (1.00, 1.40)	1.19 (1.00, 1.41)
Med-High	1.13 (0.95, 1.33)	1.16 (0.98, 1.38)
High	1.14 (0.96, 1.35)	1.17 (0.98, 1.39)
1,3-Butadiene		
Low	1.00	1.00
Medium	1.21 (1.02, 1.43)	1.23 (1.03, 1.46)
Med-High	1.18 (0.99, 1.40)	1.23 (1.04, 1.47)
High	1.22 (1.03, 1.44)	1.28 (1.08, 1.52)
Polycyclic Organic Matter		
Low	1.00	1.00
Medium	0.93 (0.78, 1.10)	0.94 (0.79, 1.12)
Med-High	1.13 (0.96, 1.34)	1.18 (1.00, 1.39)
High	1.07 (0.91, 1.27)	1.11 (0.94, 1.32)

^a^Model 1 includes air pollutant exposure variables, matching variables and census tract (random effect). *N* = 13,420

^b^Model 2 includes variables from Model 1, as well as maternal age, infant birth weight (per 100 grams), infant gender and maternal race/ ethnicity. *N* = 13,420

Results for the co-pollutant models appear in Table 3. In fully adjusted models that included benzene and 1,3-butadiene, odds of ALL remained elevated for 1,3-butadiene (medium group: OR = 1.22, 95 % CI: 1.00–1.50; medium-high group: OR = 1.28, 95 % CI: 1.00–1.63; and high exposure group: OR = 1.40, 95 % CI: 1.06–1.86). In contrast, for benzene, results were close to the null value (OR = 1.07, 0.95, and 0.91 for the medium, medium-high and high exposure groups, respectively). Similarly, in the co-pollutant model with 1,3-butadiene and POM, odds remained elevated for 1,3-butadiene but not for POM. When benzene and POM were evaluated simultaneously in the same model, odds were generally elevated but diminished in magnitude relative to the results for either pollutant alone.

**Table 3:** Association between Air Toxics and Acute Lymphocytic Leukemia in Texas using Co-Pollutant Models

Air Toxic	Model 1^a^ OR (95 % CI)	Model 2^b^ OR (95 % CI)
Benzene and 1,3-Butadiene		
Benzene		
Low	1.00	1.00
Medium	1.06 (0.87, 1.30)	1.07 (0.87, 1.31)
Med-High	0.96 (0.75, 1.22)	0.95 (0.75, 1.22)
High	0.92 (0.70, 1.22)	0.91 (0.69, 1.20)
1,3-Butadiene		
Low	1.00	1.00
Medium	1.19 (0.97, 1.45)	1.22 (1.00, 1.50)
Med-High	1.22 (0.96, 1.55)	1.28 (1.01, 1.63)
High	1.32 (1.00, 1.74)	1.40 (1.06, 1.86)
1,3-Butadiene and Polycyclic Organic Matter		
1,3-Butadiene		
Low	1.00	1.00
Medium	1.25 (1.04, 1.50)	1.26 (1.05, 1.52)
Med-High	1.17 (0.94, 1.46)	1.23 (0.99, 1.53)
High	1.20 (0.94, 1.53)	1.24 (0.97, 1.60)
Polycyclic Organic Matter		
Low	1.00	1.00
Medium	0.86 (0.71, 1.03)	0.88 (0.73, 1.06)
Med-High	1.03 (0.83, 1.28)	1.05 (0.85, 1.31)
High	0.97 (0.76, 1.24)	1.00 (0.78, 1.28)
Benzene and Polycyclic Organic Matter		
Benzene		
Low	1.00	1.00
Medium	1.21 (1.00, 1.47)	1.21 (1.00, 1.47)
Med-High	1.08 (0.87, 1.34)	1.09 (0.87, 1.35)
High	1.07 (0.84, 1.36)	1.07 (0.84, 1.37)
Polycyclic Organic Matter		
Low	1.00	1.00
Medium	0.87 (0.71, 1.05)	0.89 (0.73, 1.08)
Med-High	1.08 (0.88, 1.33)	1.12 (0.91, 1.38)
High	1.05 (0.82, 1.33)	1.10 (0.86, 1.39)

^a^Model 1 includes air pollutant exposure variables, matching variables and census tract (random effect). *N* = 13,420

^b^Model 2 includes variables from Model 1, as well as maternal age, infant birth weight (per 100 grams), infant gender and maternal race/ ethnicity. *N* = 13,420

## Discussion

We evaluated associations between residential exposures to air toxics, namely, benzene, 1,3-butadiene and POM, and ALL in children diagnosed under age 5 years in Texas from 1995 to 2011. Texas provided an unique study area to assess health effects associated with exposures to air toxics because of its extensive road traffic and congested roadways in and around large urban areas [39], abundant number of petrochemical plants [40], some of the largest petroleum refineries in the US and active seaports [14]. Moreover, the Texas Cancer Registry has more than a decade of cancer incidence data and this allowed for a population-based study with a large sample size. We found increased odds of early childhood ALL for 1,3-butadiene, benzene and POM when evaluated singly in separate models. However, in co-pollutant models, odds remained elevated only for exposure to 1,3-butadiene.

The Clean Air Act sets national air quality standards for 6 criteria air pollutants. Despite classification by the US EPA and WHO as human carcinogens, there are no mandated ambient air quality standards in the U.S. for benzene and 1,3-butadiene though their emissions are federally regulated [41]. The Texas Commission for Environmental Quality has suggested guidelines for long term (annual mean) levels in ambient air are 4.5 μg/m^3^ for benzene and 20.17 μg/m^3^ for 1,3-butadiene [42]. In our study, mean ambient air concentrations were well below these suggested levels for all time periods as indicated by Fig. 2.

The International Agency for Research on Cancer (IARC) and U.S. EPA classify 1,3-butadiene as a human carcinogen based on evidence from occupational studies [16, 43, 44]. Studies on a cohort of butadiene monomer production workers from a Texaco plant in Texas, two styrene-butadiene rubber (SBR) production cohorts and overlapping studies with added follow-up time and improved exposure assessments of the aforementioned cohorts provide evidence of higher leukemia mortality among exposed workers. In these studies (with multiple exposure metrics that included intensity, length of exposure, cumulative exposure and time period when exposures occurred), relative risks appeared dose-dependent [44]. Non-occupational studies involving 1,3-butadiene are scant. One in Canada and another in the United Kingdom documented population-based exposures and have shown increased butadiene levels in urban areas adjacent to industrial sites [16, 45, 46]. A retrospective cohort of approximately 15,000 Texas students who attended a high school that was adjacent to a SBR production facility did not find an increase in deaths as compared to the general population [16].

After adjustment for benzene (and POM), we observed positive associations between 1,3-butadiene and odds of early childhood ALL [44, 47], with the strongest associations detected for children in the highest exposure group. The two previous studies that evaluated risks of childhood leukemia with outdoor air levels of 1,3-butadiene reported similar results. In an ecologic study, for children under age 19 years at diagnosis living in Texas census tracts with the highest 1,3-butadiene levels, a rate ratio of 1.32 (95 % CI: 0.98–1.77) was detected for ALL [25]. Our results are also consistent with Heck et al. [21] who found elevated odds for ALL (OR = 1.76, 95 % CI: 1.09–2.86) associated with 1,3-butadiene exposures throughout pregnancy.

It is suggested pediatric leukemia follows Knudson’s two-stage model for carcinogenesis [48–51]. Unlike cellular origins of leukemia in adults, multipotent stem cells active in early fetal development are most likely involved in carcinogenesis [49]. *In utero* or early neonatal exposures to environmental contaminants can produce translocations and genetic alterations to hematopoietic stem cells which may be the first step in pathogenesis [2, 49, 50]. Experimental studies using rodent models suggest the mutagenic action of 1,3-butadiene epoxy metabolites on DNA is the mechanism inducing carcinogenicity. 1,3-butadiene is metabolically activated to produce reactive epoxides, diepoxybutane, epoxybutene and epoxybutanediol [16]. Epoxybutene formed in bone marrow cells and acting as an alkylating agent, may be an initiating step in hematopoietic carcinogenesis though diepoxybutane is also highly genotoxic and cross links with guanine in DNA bases [44, 52]. Moreover, polymorphisms in epoxy hydrolase or glutathione transferase may mediate genotoxic effects of butadiene exposure [44].

DNA and hemoglobin adducts could be instrumental in identifying susceptible populations at greater risk from high levels of butadiene exposures [53]. Genetic toxicology studies show higher frequency of hemoglobin adducts and HPRT variant mutations by genotype of metabolic enzymes glutathione transferase and microsomal epoxide hydrolase [44, 54]. Also, a urinary biomarker study showed quantifiable levels of 1,3-butadiene epoxide metabolites among adults with traffic-related exposures [55]. Corollary work documenting exposures to 1,3-butadiene and subsequent early genetic or non-genetic changes among young children is needed to further understand the role of this air toxic on early childhood leukemogenesis.

Ambient air contains a mixture of contaminants, particularly in areas with multiple pollution sources, which can vary geographically and over time [53, 56]. The mixture of air toxics makes it difficult to differentiate the impacts of multiple pollutants in epidemiologic studies. Our results suggest the associations we detected in single pollutant models for benzene and POM were likely the result of confounding due to 1,3-butadiene. Other epidemiologic studies are needed to confirm our findings. However, our results are consistent with previous occupational cohort studies showing leukemia risks associated with butadiene exposures are confounded by co-pollutant exposures. Similar to our findings, Macaluso et al. showed leukemia risk associated with benzene is negated after controlling for butadiene and styrene [57]. And, analogous to our results with benzene and POM, styrene and dithiocarbamates (which are present in SBR production but not monomer production) show positive associations with leukemia in single pollutant models but not in co-pollutant models with butadiene [44].

Primary among the strengths of this study is the focus on early childhood ALL. In children, ALL incidence is greatest before age 5 [50]. Yet, relatively few investigations have examined exposure to air toxics in study populations of this age range [21, 24, 28, 58]. Differential patterns of risk and strength of point estimates on age stratification suggest divergent etiological pathways based on age of diagnosis [24, 58]. Children with leukemia onset under age 5 years likely represent a sub-population with *in utero* or early infant exposures or an underlying biological susceptibility that increases risk of developing hematopoietic cancers [48, 50, 53].

Additional strengths of the study include use of birth records to identify controls representative of the source population [59]. Employing a population-based study design, we were able to construct exposure estimates for our entire study population, making our study less prone to selection bias as might be the case when participants are selected based on residential proximity to existing air monitoring stations [21, 28] or participation is restricted to parents who had not moved after childbirth [27]. Further, our linkage of birth records to cancer registry data provided individual-level confounder information. These data were relatively complete for variables in our investigation and validity studies have found birth certificate data have high rates of correspondence with the same information present on medical records [60–64] or available from data gathered in epidemiologic studies [61, 65]. Moreover, we had information on tobacco use during pregnancy, which is missing is other studies of childhood leukemia associated with air toxic exposures [20, 21, 23–26, 28, 58].

Our study limitations include use of U.S. EPA NATA modeled data serving as a surrogate for personal exposure, which is influenced by air pollutant levels in multiple outdoor and indoor environments and time spent in each microenvironment [66]. Lacking available personal- or area-level air pollutant measurements for a majority of census tracts in the U.S., NATA provides useful surrogate data for studies that examine health effects associated with ambient exposures. However, we note that the 1996 POM levels may be overestimated due to methods used to categorize PAH emissions from stack sources in the first NATA release [67] and that modelled values for benzene in 2002 may be moderately overestimated based on EPA assessments to monitored data [68]. In general, comparisons of monitored and modelled air pollutant levels show agreement (especially for benzene and 1,3-butadiene), although modelled estimates tend to underestimate air monitoring data [68–72]. Payne-Sturges et al. similarly reported good agreement between NATA data and personal exposures to benzene (1,3-butadiene and PAHs were not evaluated) [73].

Improvements in emissions, reporting, laboratory methods and sensor technology may also influence differences in air pollutant levels across the NATA releases. NATA data were only available for 1996, 1999, 2002 and 2005 (the recently issued data release for 2011 was not available when the study was conducted) and this may have introduced error in the exposure assessment. However, our sensitivity analysis restricting the study population to cases and controls born within one year of NATA release produced similar results as compared to the full study population and, hence, this limitation does not appear to have adversely affected study findings. Third, while we used maternal residence at birth to link to the modeled air toxics data, we lacked information about mobility during pregnancy or after birth. Nevertheless, Lupo et al. [74] found moving residences during pregnancy among Texas women did not significantly impact classification of benzene exposure. Our ability to examine disease risks by subtype or age at diagnosis was constrained by power considerations. AML and ALL likely have different causal pathways given geographic, demographic and diagnostic age differences among children and adults. Moreover, Cheng et al. found cumulative exposures to butadiene increased lymphoid neoplasms while peak exposures were associated with myeloid neoplasms [44] in an SBR occupational cohort. With sufficient AML cases in future, we could expanded our study to examine risks by histological subtype allowing the potential to determine if these findings translate to childhood leukemia risk associated with ambient residential exposures.

## Conclusions

This study is among relatively few investigations that have examined risk of ALL relative to ambient exposures to benzene, 1,3-butadiene and POM among children less than 5 years. While we observed positive associations between all three air toxics and early childhood ALL in single pollutant models, our co-pollutant models suggest that the results for benzene and POM were likely confounded by 1,3-butadiene exposure. The consistent increase in ALL (approximately 25 %) associated with medium to high quartile ambient butadiene levels compared to the lowest quartile in this study suggest in utero exposure to butadiene may play a more important role than benzene in early childhood leukemia etiology. More work is needed to confirm these findings in other studies.

## Abbreviations

ALL: acute lymphocytic leukemia; AML: acute myeloid leukemia; ASPEN: Assessment System for Population Exposure Nationwide; CI: confidence interval; DNA: deoxyribonucleic acid; IARC: International Agency for Research on Cancer; ICD-O-3: International Classification of Diseases for Oncology, 3^rd^ edition; NATA: US EPA National-Scale Air Toxics Assessment; OR: odds ratio; PAH: polycyclic aromatic hydrocarbons; POM: polycyclic organic matter; SBR: styrene-Butadiene rubber; SEER: surveillance, epidemiology and end results; TCR: Texas Cancer Registry; TX DSHS: Texas Department of State Health Services; U.S.: United States of America; US EPA: United States Environmental Protection Agency; WHO: World Health Organization.

## Additional files

Additional file 1: Figure S1. Benzene Ambient Air Levels in Texas by NATA Year. Benzene Ambient Air Levels in Texas by NATA Year. Map illustrating spatial distribution of ambient air benzene concentrations for all census tracts in Texas by NATA years included in our study. Figure S1: Benzene Ambient Air Levels in Texas by NATA year. Exposure groups: Low (0-24^th^ percentile); Medium (25-49^th^ percentile); Medium-High (50–74^th^ percentile); High (75–100^th^ percentile) based on distribution among controls. Boundary lines for census tracts were removed for the Medium, Medium-High, and High groups and graduated color used instead to improve readability of maps and spatial distribution of exposure quartiles. (PDF 1808 kb) Additional file 2: Figure S2. POM Ambient Air Levels in Texas by NATA Year. POM Ambient Air Levels in Texas by NATA Year. Map illustrating spatial distribution of ambient air POM concentrations for all census tracts in Texas by NATA years included in our study. Figure S2: POM Ambient Air Levels in Texas by NATA year. Exposure groups: Low (0–24^th^ percentile); Medium (25–49^th^ percentile); Medium-High (50–74^th^ percentile); High (75–100^th^ percentile) based on distribution among controls. Boundary lines for census tracts were removed for the Medium, Medium-High, and High groups and graduated color used instead to improve readability of maps and spatial distribution of exposure quartiles. (PDF 1816 kb)
