# Supplementary figures and images for: Air toxics and early childhood acute lymphocytic leukemia in Texas, a population based case control study
Source: Environ Health. 2016 Jun 14;15:70. doi: 10.1186/s12940-016-0154-8 (PMC4908700; doi:10.1186/s12940-016-0154-8)

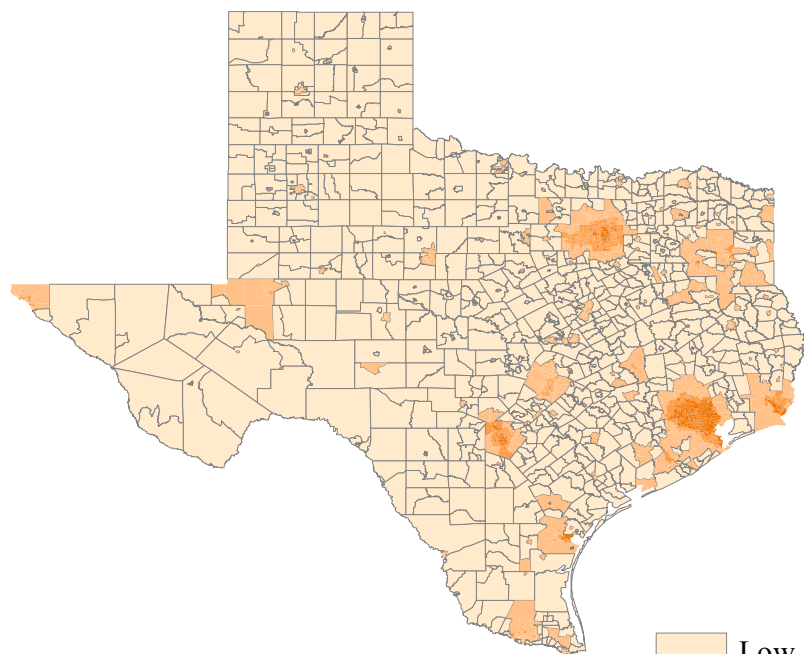

**NATA 1996**

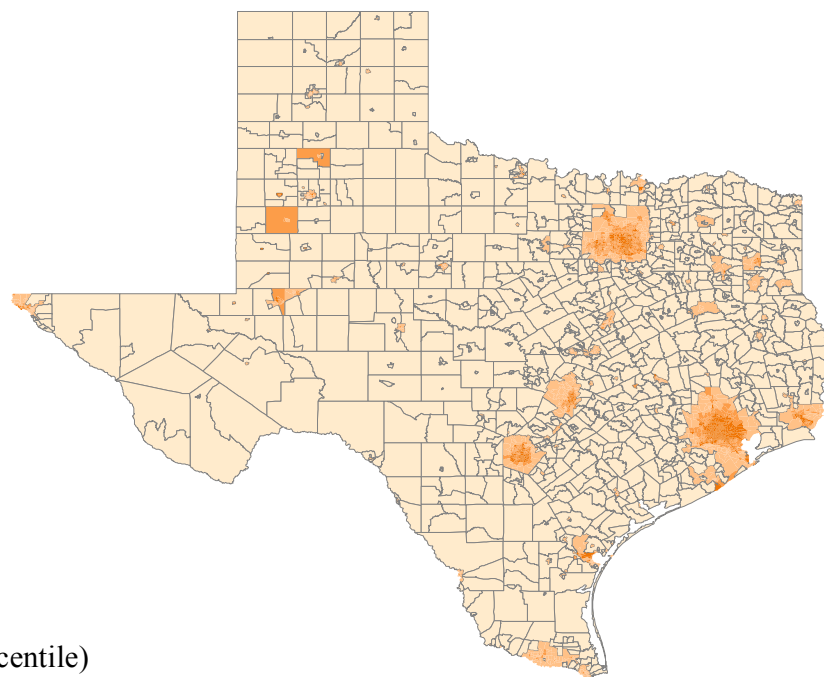

**NATA 1999**

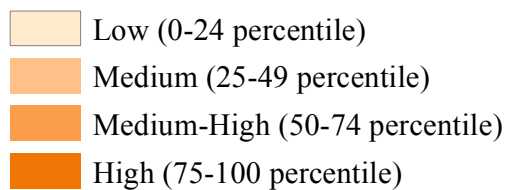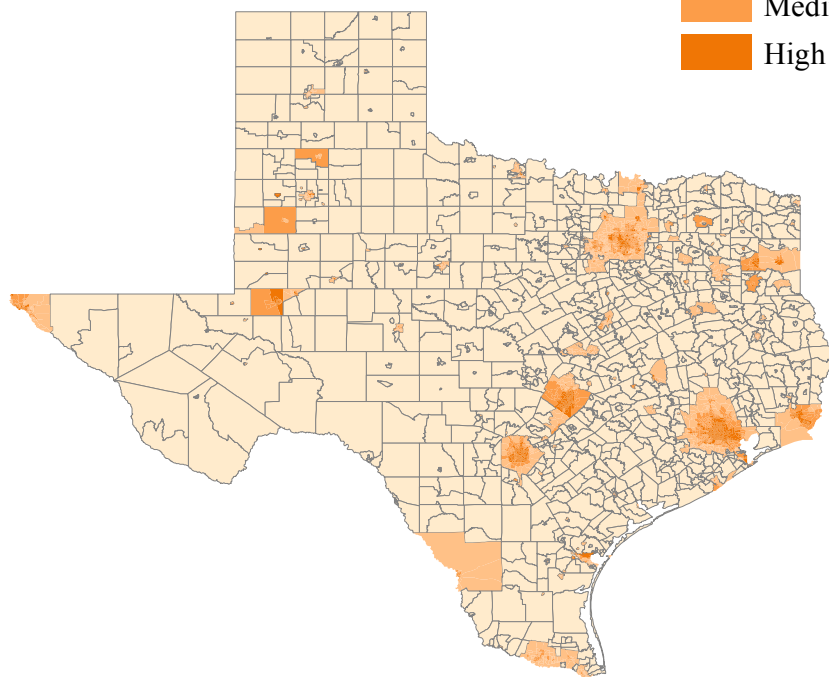

**NATA 2002**

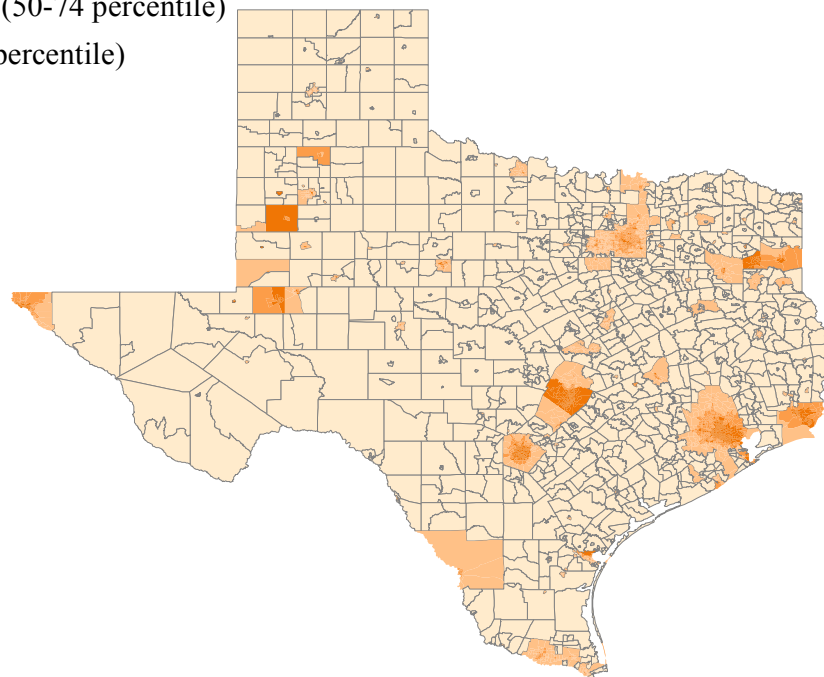

**NATA 2005**

Supplement: Additional file 1: Figure S1. — Benzene Ambient Air Levels in Texas by NATA Year. Benzene Ambient Air Levels in Texas by NATA Year. Map illustrating spatial distribution of ambient air benzene concentrations for all census tracts in Texas by NATA years included in our study. Figure S1: Benzene Ambient Air Levels in Texas by NATA year. Exposure groups: Low (0-24th percentile); Medium (25-49th percentile); Medium-High (50–74th percentile); High (75–100th percentile) based on distribution among controls. Boundary lines for census tracts were removed for the Medium, Medium-High, and High groups and graduated color used instead to improve readability of maps and spatial distribution of exposure quartiles. (PDF 1808 kb) [file 12940_2016_154_MOESM1_ESM.pdf]

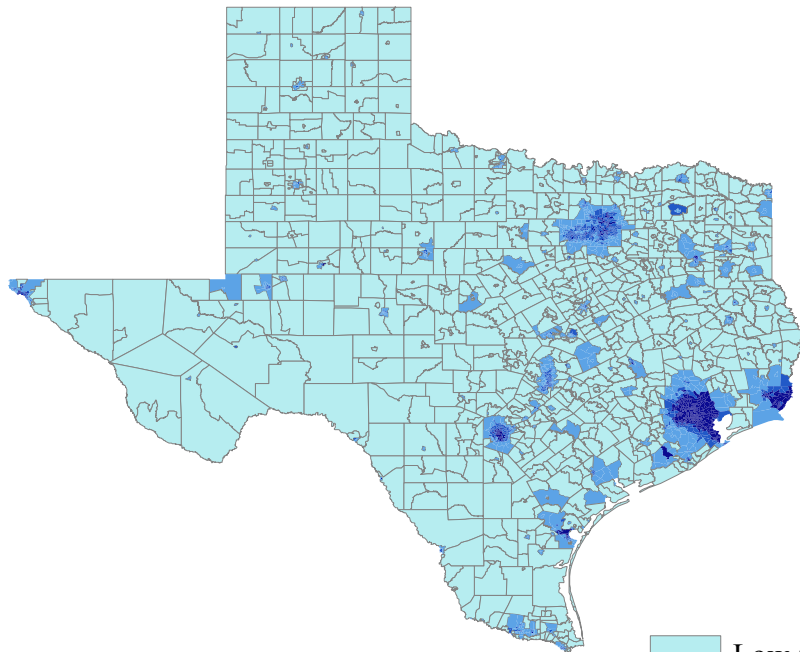

**NATA 1996**

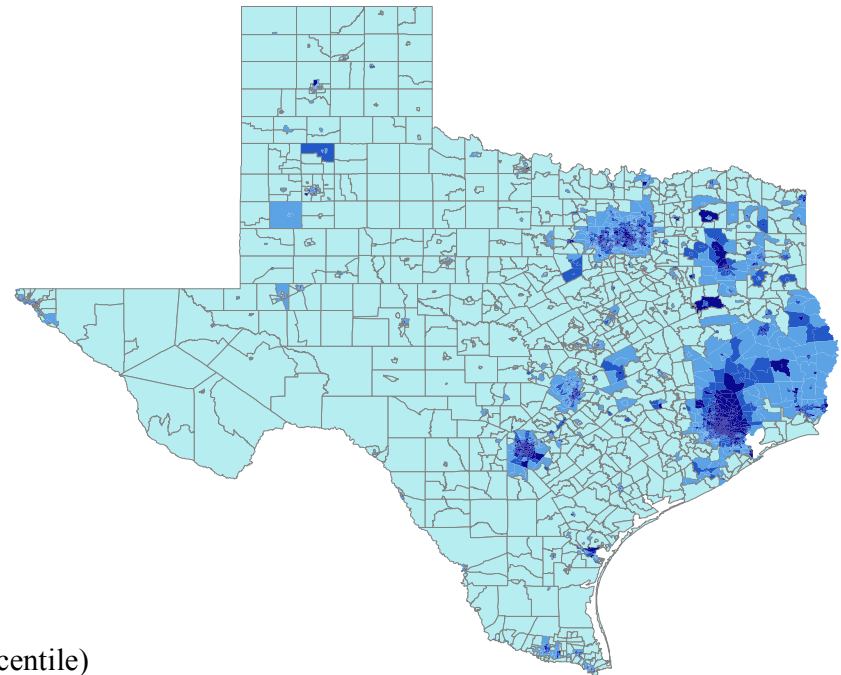

**NATA 1999**

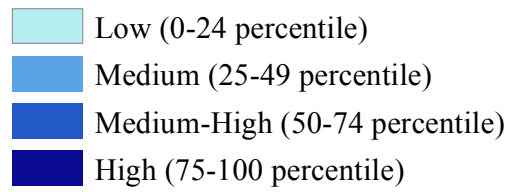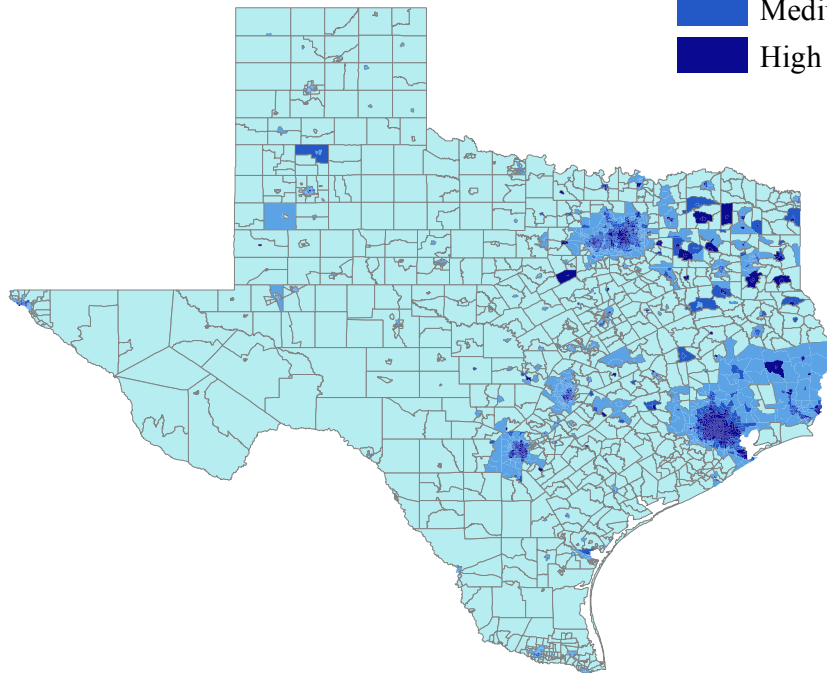

**NATA 2002**

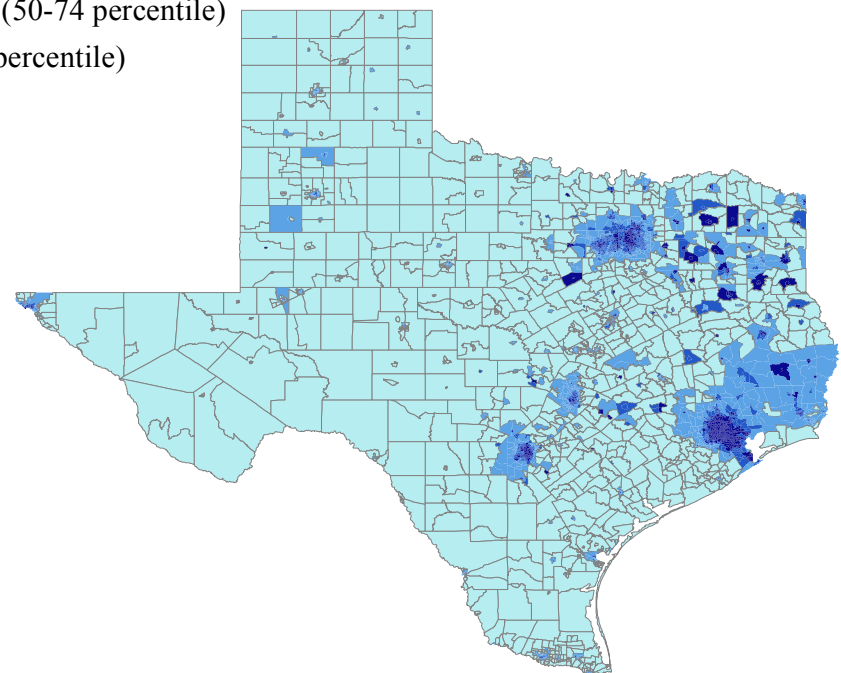

**NATA 2005**

Supplement: Additional file 2: Figure S2. — POM Ambient Air Levels in Texas by NATA Year. POM Ambient Air Levels in Texas by NATA Year. Map illustrating spatial distribution of ambient air POM concentrations for all census tracts in Texas by NATA years included in our study. Figure S2: POM Ambient Air Levels in Texas by NATA year. Exposure groups: Low (0–24th percentile); Medium (25–49th percentile); Medium-High (50–74th percentile); High (75–100th percentile) based on distribution among controls. Boundary lines for census tracts were removed for the Medium, Medium-High, and High groups and graduated color used instead to improve readability of maps and spatial distribution of exposure quartiles. (PDF 1816 kb) [file 12940_2016_154_MOESM2_ESM.pdf]
